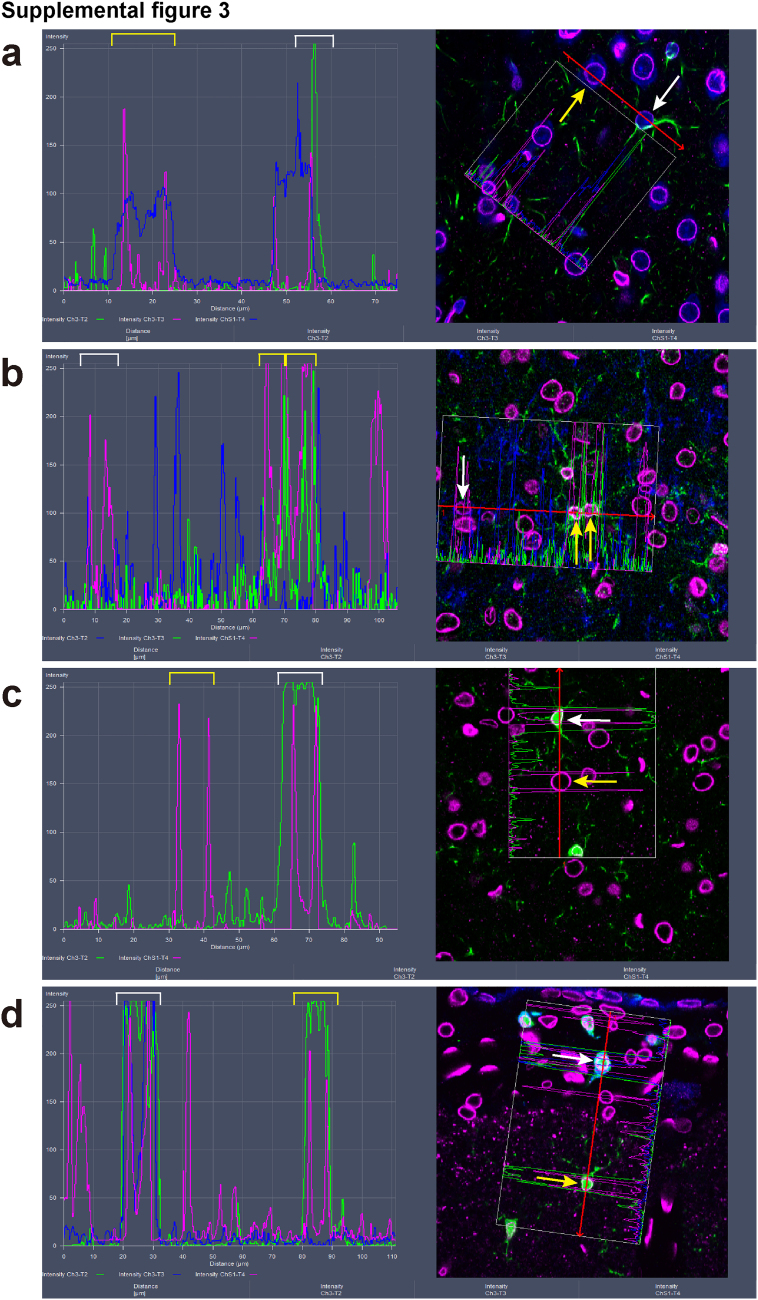# Differential expression of nuclear lamin subtypes in the neural cells of the adult rat cerebral cortex

**DOI:** 10.1016/j.ibror.2018.11.001

**Published:** 2018-11-05

**Authors:** Yasuharu Takamori, Yukie Hirahara, Taketoshi Wakabayashi, Tetsuji Mori, Taro Koike, Yosky Kataoka, Yasuhisa Tamura, Shuji Kurebayashi, Kiyoshi Kurokawa, Hisao Yamada

**Affiliations:** a Department of Anatomy and Cell Science, Kansai Medical University, Osaka, Japan; b Faculty of Medicine, Tottori University, Tottori, Japan; c Laboratory for Cellular Function Imaging, RIKEN Center for Biosystems Dynamics Research, Kobe, Japan; d Multi-Modal Microstructure Analysis Unit, RIKEN-JEOL Collaboration Center, Kobe, Japan; e Department of School Education Research, Shizuoka University, Shizuoka, Japan; f Department of Human Health Science, Osaka international University, Osaka, Japan

**Keywords:** Lamins, Neurons, Glial cells, Adult rat, Cerebral cortex, Immunohistochemistry

## Abstract

Lamins are type V intermediate filament proteins that are located beneath the inner nuclear membrane. In mammalian somatic cells, LMNB1 and LMNB2 encode somatic lamins B1 and B2, respectively, and the LMNA gene is alternatively spliced to generate somatic lamins A and C.

Mutations in lamin genes have been linked to many human hereditary diseases, including neurodegenerative disorders. Knowledge about lamins in the nervous system has been accumulated recently, but a precise analysis of lamin subtypes in glial cells has not yet been reported. In this study we investigated the composition of lamin subtypes in neurons, astrocytes, oligodendrocyte-lineage cells, and microglia in the adult rat cerebral cortex using an immunohistochemical staining method. Lamin A was not observed in neurons and glial cells. Lamin C was observed in astrocytes, mature oligodendrocytes and neurons, but not observed in oligodendrocyte progenitor cells. Microglia also did not stain positive for lamin C which differed from macrophages, with lamin C positive. Lamin B1 and B2 were observed in all glial cells and neurons. Lamin B1 was intensely positive in oligodendrocyte progenitor cells compared with other glial cells and neurons. Lamin B2 was weakly positive in all glial cells compared to neurons. Our current study might provide useful information to reveal how the onset mechanisms of human neurodegenerative diseases are associated with mutations in genes for nuclear lamin proteins.

## Highlights

• This work demonstrates the expression patterns for lamin subtypes in neural cells in the adult rat cerebral cortex.
• This work demonstrates suitable fixative conditions of multiple immunostaining systems for lamins and cell-type specific marker proteins.
• Neural cells in the adult cerebral cortex showed heterogeneous lamin antibody labeling patterns.

## Introduction

Nuclear lamins are type V intermediate filament proteins in the nucleus that form a nuclear lamina, which is a filamentous or meshwork structure underlying the inner nuclear membrane (Burke and Stewart, 2013; Zuela et al., 2012). Lamins were originally identified as lamins A, B, and C (Gerace and Blobel, 1980). Lamins A and C, as well as two other variants, AD10 and sperm-specific C2, are classified as A-type lamins and are encoded by the *LMNA* gene, which produces each specific subtype through alternative splicing. Three different B-type lamin proteins are encoded by two genes (B1 by *LMNB1* and B2 and sperm-specific B3 by *LMNB2*). The nuclear lamina associates with other nuclear envelope proteins and plays numerous roles, including maintaining the nuclear shape and structure, assembly and disassembly of the nucleus, heterochromatin organization, transcriptional regulation, and other nuclear functions (Burke and Stewart, 2013; Zuela et al., 2012; Simon and Wilson, 2013). Gene mutations for lamins and lamin-associated proteins can cause diverse human diseases called “laminopathies” or “nuclear envelopathies”, such as cardiac and muscular dystrophy, lipodystrophy, and premature ageing disorder (Schreiber and Kennedy, 2013).

Previous studies revealed that B-type lamins are expressed in most or all cell types, whereas A-type lamins are not expressed in immature cells during early developmental stages in mice (Röber et al., 1989; Burke and Stewart, 2013; Zuela et al., 2012). In adult tissues, lamin A/C is not expressed in immature cells but is observed in fully differentiated cells, while lamin B1 is expressed abundantly in immature cells when compared with differentiated cells in some types of epithelia and other tissues. Lamin B2 is ubiquitously expressed when compared with lamin A/C and B1. These reports suggest that the expression of each lamin subtype is regulated in tissue-specific, as well as cell-type specific manners. (Broers et al., 1997; Coates et al., 1996; Willis et al., 2008; Takamori et al., 2007).

Lamins in the nervous system and in other tissues have been investigated recently. A unique mutation in the *LMNA* gene leads to autosomal recessive axonal Charcot-Marie-Tooth disease type 2B, which is characterized by the loss of peripheral nerve myelination associated with wasting and weakness in all four limbs ((Schreiber and Kennedy, 2013; De Sandre-Giovannoli et al., 2002; Tazir et al., 2013). Duplication of the *LMNB1* gene, which causes increased expression of lamin B1, is associated with autosomal dominant leukodystrophy (ADLD), a rare adult-onset disease characterized by progressive myelin loss in the central nervous system (Burke and Stewart, 2013; Padiath et al., 2006; Zuela et al., 2012). Analysis of an in vitro culture system and transgenic mice revealed that overexpression of lamin B1 in cells of the oligodendrocyte cellular lineage suppresses differentiation and myelin formation and that microRNA-23 (miR-23), an abundant miRNA in oligodendrocytes, represses the expression of lamin B1 and leads to increased myelination and enhanced oligodendrocyte differentiation (Lin and Fu, 2009; Heng et al., 2013; Lin et al., 2013, 2014). A series of experiments using transgenic mice revealed that knockout or partial deletion of lamin B1, B2 or both in the brain results in improper brain development with abnormalities of nuclear shape, spindle apparatus orientation, cell cycle regulation and neuronal migration (Vergnes et al., 2004; Coffinier et al., 2010, 2011; Jung et al., 2013; Lee et al., 2014). In the brain, lamin C encoded by *LMNA* gene is a major A-type lamin, and the levels of lamin A mRNA are regulated specifically by brain-specific microRNA miR-9 (Jung et al., 2012, 2014; Zuela et al., 2012).

As lamins are predominantly localized to the inner nuclear membrane, we used an anti-lamin B1 antibody as a nuclear membrane marker combined with BrdU labelling to determine the positions of the cell nuclei in oligodendrocytes and neurons in the rat cerebral cortex (Kataoka et al., 2006; Tamura et al., 2007). Immunoreactivity for lamin A/C was diminished during adult neurogenesis, and lamin B1 was increased transiently in neuronal progenitor cells that were positive for PSA-NCAM and doublecortin and were localized in two neurogenic regions of the mammalian brain, namely, the subventricular zone of the lateral ventricle and the subgranular zone of the dentate gyrus (Takamori et al., 2007, 2014). All neurons in the adult rat retina were positive for lamin B1 and B2, while some kinds of retinal neurons were negative for lamin A, and photoreceptor cells were negative for lamin A and C (Wakabayashi et al., 2011). However, many types of glial cells are distributed in the brain, and detailed analyses of lamin subtypes in each glial cell type are not yet reported. The analysis of cell-type specific expression of lamin subtypes in the glial cells may help in understanding the functional differences of lamin subtypes, as well as in understanding the pathogenesis of nuclear lamina-associated neurodegenerative diseases.

We previously noticed that most cell nuclei in the brain parenchyma stained with antibodies recognizing both lamin A and C but were not stained with an anti-lamin A-specific antibody. We speculated that most cells in the brain only express lamin C and not lamin A. However, immunostaining using anti-lamin antibodies was not stable in formaldehyde fixation, and use of anti-lamin antibodies was only possible in methanol-acetone fixation, which made detailed immunohistochemical analysis using antibodies against lamin-subtypes and cell-type specific marker proteins quite difficult.

In this study, we have investigated the composition of lamin subtypes in neurons, astrocytes, oligodendrocyte-lineage cells, and microglia in the adult rat cerebral cortex. We improved and performed multiple immunostaining analyses by using antibodies against each lamin subtype and cell-type specific marker proteins through use of a confocal laser microscope.

## Methods

### Animals

Adult male Wistar/ST rats (8 weeks old; Nippon SLC, Hamamatsu, Japan) were purchased from Shimizu Laboratory Supplies (Kyoto, Japan) and used for all experiments. The Animal Ethics Committee of Kansai Medical University approved all experimental protocols, and all studies were performed in accordance with the Principles of Laboratory Animal Care (NIH publication No. 85-23, revised 1985)

### Tissue preparation

The animals were anaesthetized by intraperitoneal injection with a mixture of medetomidine (0.375 mg/kg), midazolam (2 mg/kg) and butorphanol (2.5 mg/kg) in phosphate-buffered saline (PBS). We used three different fixation procedures based on the immunohistochemical conditions recommended for each primary antibody. To perform immunohistochemistry for lamin A, C and B2, animals were transcardially perfused with 0.1 M PBS at pH 7.4 under deep anaesthesia, followed by a fixative containing 4% formaldehyde and 0.2% picric acid in 0.1 M phosphate buffer (pH 7.4). The brains were dissected and post-fixed for 24 h in the same fixative at 4 °C. After fixation, brains were cryoprotected in 10% sucrose solution (in 0.1 M phosphate buffer, pH 7.4) for 24 h followed by incubation in 20% sucrose solution (in 0.1 M phosphate buffer, pH 7.4) for 24 h. For cryosectioning, fixed brain tissues were embedded in Tissue-Tek O.C.T. compound (Sakura Finetek Japan, Tokyo, Japan) and snap-frozen with dry ice. Coronal sections (30 μm thickness) were obtained with a cryostat. These sections were stored at 4 °C in PBS and used for free-floating immunohistochemistry. For antigen retrieval, fixed sections were boiled in 10 mM sodium citrate buffer (pH 6.0) for 30 min. To perform immunohistochemistry for lamin B1, fixative containing 1% formaldehyde and 0.05% picric acid in 0.1 M phosphate buffer (pH 7.4) was used for mild formaldehyde fixation. Antigen retrieval was not performed in this condition. Methanol and acetone fixation were performed using our previous protocol (Takamori et all., 1997). The animals were sacrificed by cervical dislocation under deep anaesthesia. The brains were dissected and snap-frozen with CO_2_ gas. Coronal sections at a 10 μm thickness were cut using a cryostat, collected on glass slides and air-dried at room temperature for 1 h. Subsequently, the sections were fixed in cold methanol at −20 °C for 10 min, followed by three dips in acetone at 4 °C (5 s each) and air-dried for 5 min.

### Immunohistochemistry

Immunohistochemistry was performed on tissues from a total of 18 rats fixed with three different fixation methods as mentioned above. Brain coronal sections were obtained by free-floating sections from positions at approximately bregma −1.5 mm to bregma +4.5 mm (Paxinos and Watson, 1986). Ten sections per animal were chosen for immunohistochemistry. Primary antibodies used are summarized in Table 1. Free-floating sections were incubated with primary antibodies in PBST (PBS with 0.03% Triton X-100) at 4 °C for 24 h. Then, sections then were rinsed in PBST two times for 15 min at room temperature and incubated with Cy2-, Cy3- or Cy5-labelled donkey secondary antibodies specific to the appropriate animal species (Jackson Immuno-Research, West Grove, PA, USA; 1:200) in PBST at 4 °C for 3 h. Alexa Fluor 488-, and 555-labelled donkey secondary antibodies (1:400, Invitrogen/Molecular Probes, Carlsbad, CA, USA) were used in some cases. Sections were rinsed in PBST two times and then mounted with a medium containing 100 mM DTT, 5 μg/ml Hoechst dye 33258 (Nacalai Tesuque Inc., Kyoto, Japan), and 50% glycerol in PBS at pH 7.4. Some sections were counterstained with TOPRO-3 (Molecular Probes, Eugene, OR, USA; 1:1000) for nucleic acid detection. Fluorescently immunostained sections were observed and photographed with a confocal laser microscope (model LSM510-META; Carl Zeiss, Oberkochen, Germany). The fluorescent signal intensity was measured with an imaging browser for micrographs (ZEN, Carl Zeiss, RRID:SCR_013672). The area from cortical layer II to layer V in the parietal and temporal lobes of the cerebral cortex of the 10 chosen sections was examined with a confocal laser microscope (LSM 510, Carl Zeiss). Fluorescent images were acquired as single optical sections at 1-μm intervals to detect the borders of the nucleus and cytosol clearly, which allowed identification of the cell-specific expression of lamin. The goat polyclonal anti-lamin B1 antibody (Santa Cruz Biotechnology) recognizes lamin B1 from extracts of primary mouse embryonic fibroblasts but does not recognize lamin B2 or mutant lamin B1 fused to beta-geo, which lacks 273 amino acids at its carboxy-terminus (Vergnes et al., 2004). Therefore, we used this antibody as an anti-lamin B1 antibody as previously reported (Vergnes et al., 2004; Takamori et al., 2007, 2014). Specificity of other anti-lamin antibodies had already been confirmed by two-dimensional immunoblotting in previous studies (Machiels et al., 1995; Lehner et al., 1986), as well as Western blot analysis and peptide neutralization experiments in our previous studies (Wakabayashi et al., 2011). The original TIFF files were imported into Photoshop software (Adobe Systems, San Jose, CA, USA) to prepare the images for publication; all images were processed, adjusted for brightness and contrast, and resized to 300 dpi using this application. The level of immunoreactivity observed is indicated as follows for all results: (++) = highly positive; (+) = positive; (–) = negative (Table 2).

**Table 1:** List of antibodies used as marker.

Antibody	Immunogen	Manufacture and catalog number, clone	Species	Dilution	Cell type specificity
CD68	Rat spleen cells (Dijkstra et al., 1985)	BMA Biomedicals AG, Augst, Switzerland, T-3003, clone ED1	Mouse monoclonal IgG_1_	1/100	macrophage
GFAP	GFAP from pig spinal cord (Debus et al., 1983)	Sigma-Aldrich, St. Louis, MO, G3893, clone G-A-5	Mouse monoclonal IgG_1_	1/200	astrocyte
GFAP	GFAP from human brain	Sigma-Aldrich, G9269	Rabbit polyclonal	1/200	astrocyte
GFAP	GFAP from cow spinal cord	Dako-Japan, Kyoto, Japan, Z0334	Rabbit polyclonal	1/200	astrocyte
GST-pi	Human (GST)-pi, 5-210aa.	BD Pharmingen, San Diedo, CA, USA, 610718, clone 3/GST-pi	Mouse monoclonal IgG_1_	1/200	oligodendrocyte (mature)
Iba1	Iba1 (human, mouse, rat), C-terminus	Wako Pure Chemical Industries, Osaka, Japan. No. 019-19741	Rabbit polyclonal	1/200	microglia/ macrophage
Iba1	Human Iba1, 135-147aa.	Abcam plc, Cambridge, UK, ab5076	Goat polyclonal	1/100	microglia/ macrophage
NG2	Cell line expressing a truncated form of NG2 chondroitin sulfate proteoglycan	Millipore Bioscience Research Reagents, Temecula, CA. Mab5384, clone 132.39	Mouse monoclonal IgG_1_	1/100	oligodendrocyte (immature)
Nestin	Rat nestin, 544-776 aa.	R&D systems Inc, Minneapolis, MN, USA, AF2736	Goat polyclonal	1/200	blood cell
NeuN	Purified cell nuclei from mouse brain (Mullen et al., 1992). This antibody recognizes Fox-3/Rbfox-3 preferentially (Kim et al., 2009)	Millipore Bioscience Research Reagents, MAB377, clone A60	Mouse monoclonal IgG_1_	1/100	neuron
OLIG2	Mouse OLIG2, C-terminus	Santa Cruz Biotechnology, Santa Cruz, USA, sc-19969, C-17	Goat polyclonal	1/100	oligodendrocytes (immature/mature)
RECA-1	Stromal cells from rat lymph node (Duijvestijn et al., 1992)	Serotec, MCA970R, clone HIS52	Mouse monoclonal IgG_1_	1/200	blood cell
RIP	Rat olfactory bulb extract (Friedman et al., 1989). This antibody recognize 2,3′-cyclic nucleotide 3′-phosphodiesterase preferentially (Watanabe et al., 2006)	Millipore Bioscience Research Reagents, MAB1580, clone NS-1	Mouse monoclonal IgG_1_	1/1000	oligodendrocyte (mature)
Vimentin	Vimentin purified from pig eye lens (Osborn et al., 1984)	Sigma-Aldrich, V6389, clone V9	Mouse monoclonal IgG_1_	1/100	blood cell/ meningeal cell

GFAP: Glial fibrillary acidic protein, GST-pi :glutathione-S-transferase-pi.

**Table 2:** List of lamin antibodies.

Antibody	Immunogen	Manufacture and catalog number	Species	Fixative	Dilution
Lamin A	Human lamin A, 598–611aa.	Sigma-Aldrich, L1293	Rabbit polyclonal	4%FA-H	1/100
Lamin A	Human lamin C, 598–611aa.	Merck Millipore, MAB 3540, clone 133A2	Mouse monoclonal, IgG	Mt-Ac	1/100
Lamin C	Human lamin C, 565–572aa.	Acris, Rockville, MD, USA, BP4505S	Rabbit polyclonal	4%FA-H	1/100
Lamin B1	Rat lamin B1, whole molecule	Merck Millipore, MAB 3213, clone 119D5-F1	Mouse monoclonal, IgG	Mt-Ac	1/40
Lamin B1	Human lamin B1, C-terminus	Santa Cruz Biotechnology, sc-6216, C-20	Goat polyclonal	1%FA	1/100
Lamin B2	Extracts from chicken nuclei	Invitrogen Corporation/Zymed, Waltham, MA USA, 33-2100, clone E-3	Mouse monoclonal, IgG_1_κ	4%FA-H	1/100

1%FA: 1% formaldehyde, 4%FA-H: 4% formaldehyde combined with heat treatment (antigen retrieval), Mt-Ac: methanol-acetone.

## Results

### Glial cells and neurons in the cerebral cortex did not express lamin A

**Fig. 1:**
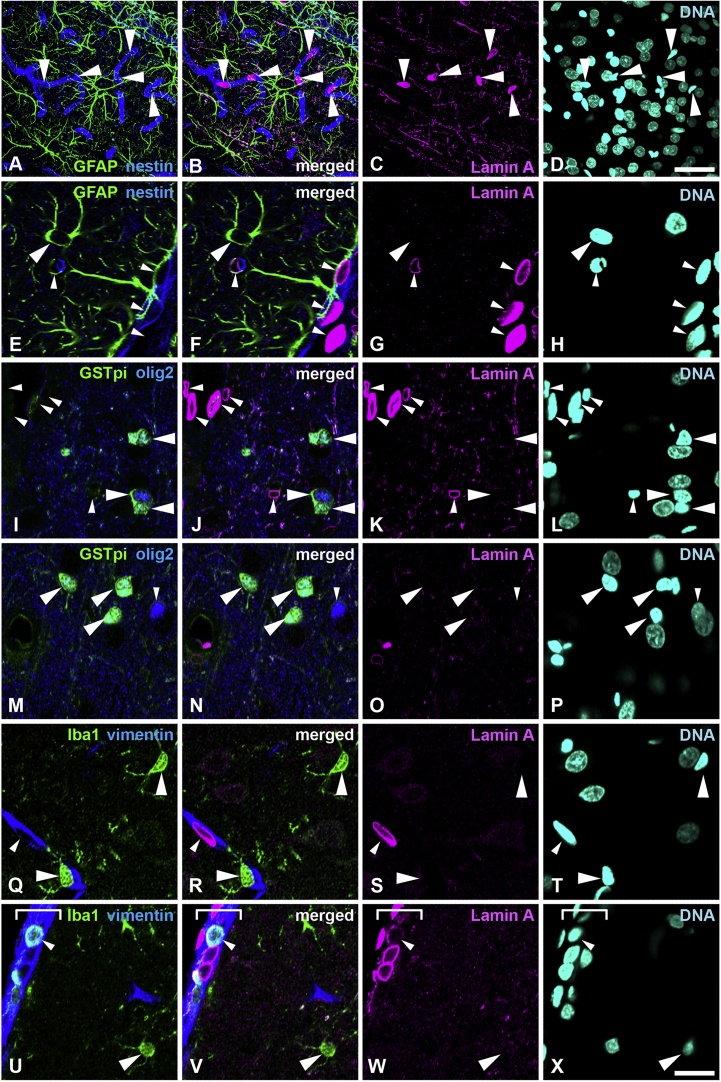
Distribution of lamin A in the cerebral cortex. A–H, Sections were stained with antibodies against lamin A (magenta), GFAP (green) and nestin (blue). A–D, Low-power field. Large arrowheads indicate lamin A-positive capillary wall cells. Twenty section images with 1-μm intervals were stacked by maximum projections. E–H, Large arrowhead indicates an astrocyte. Small arrowheads indicate capillary wall cells. I–P, Sections were stained with antibodies against lamin A (magenta), GST-pi (green) and olig2 (blue). I–L, Large arrowhead indicates mature GST-pi- and olig2-positive oligodendrocytes. Small arrowheads indicate capillary wall cells. M–P, Large arrowhead indicates GST-pi- and olig2-positive mature oligodendrocyte. Small arrowheads indicate GST-pi-negative and olig2-positive oligodendrocyte progenitor cells. Q–X, Sections were stained with antibodies against lamin A (magenta), Iba-1 (green), and vimentin (blue). Small arrowheads in Q–T indicate capillary wall cells, light blue signal shows Iba-1 positive macrophages (small arrowheads in U–X). The meninges were identified using brightfield imaging and shown with a white line bracket. Specimens were fixed using 4% formaldehyde combined with heat treatment. DNA was stained with Hoechst 33258 (cyan). Scale bar, 40 μm in A–D, 20 μm in E–X. Images of single optical sections (E-X (For interpretation of the references to colour in this figure legend, the reader is referred to the web version of this article).

**Fig. 2:**
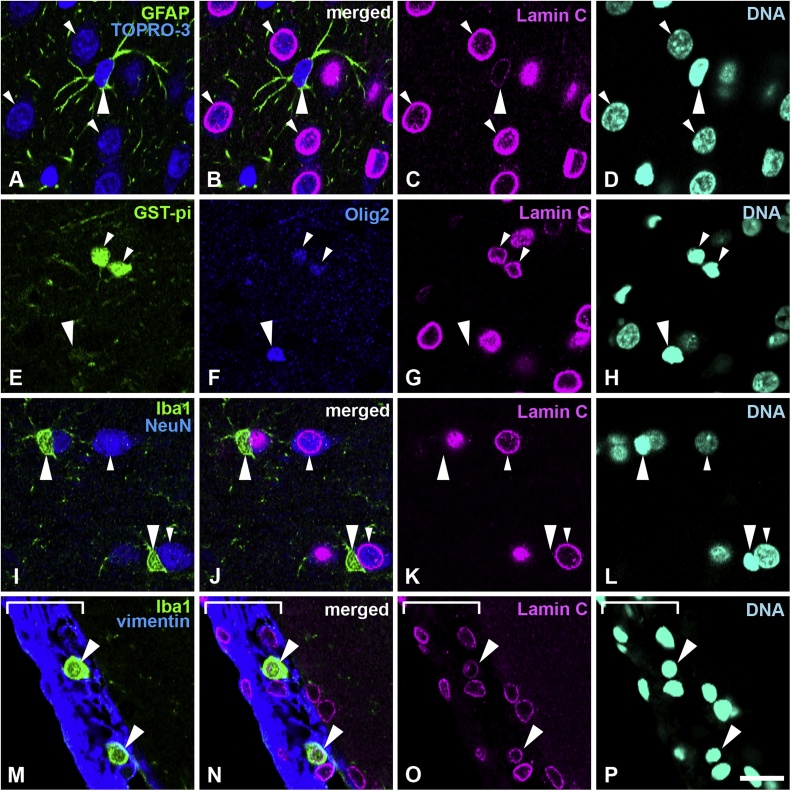
Distribution of lamin C in the cerebral cortex. A–D, Sections were stained with antibodies against lamin C (magenta) and GFAP (green) and counterstained with TOPRO-3 for nucleic acid detection (blue). Large arrowheads indicate astrocytes. Small arrowheads indicate neurons; these are further indicated with cytoplasm that is stained with TOPRO-3. E–H, Sections were stained with antibodies against GSTpi (green) and Olig2 (blue). Large arrowheads indicate an oligodendrocyte progenitor cell. Small arrowheads indicate mature oligodendrocytes. I–L, Sections were stained with antibodies against Iba-1 (green) and NeuN (blue). Large arrowheads indicate microglia. Small arrowheads indicate neurons. Note that microglia are negative for lamin C. M–P, Sections were stained with antibodies against Iba-1 (green) and vimentin (blue). Large arrowheads indicate macrophages in the meninges. The meninges are indicated with a white line bracket. Specimens were fixed using 4% formaldehyde combined with heat treatment. DNA was stained with Hoechst 33258 (cyan). Scale bar, 20 μm. Images of single optical sections (A–P) (For interpretation of the references to colour in this figure legend, the reader is referred to the web version of this article).

We used antibodies against lamin A and C that are specific to their C-termini to distinguish these two splicing variants. Sections of cortex were stained with a rabbit anti-lamin A antibody (L1293) provided by Sigma-Aldrich Co. and for several cell-type specific markers (Fig. 1). Specimens were fixed using 4% formaldehyde combined with heat treatment. Lamin A was not observed in any of the cells in the cerebral cortex except for capillary wall cells including vascular endothelial cells and pericytes, which were nestin-positive and demonstrated squamous epithelial morphology using brightfield microscopy that were positive for nestin (Fig. 1A–D, large arrowheads, and Fig. 1E–H, small arrowheads). Astrocytes were recognized with positive staining for glial fibrillary acidic protein (GFAP) (Fig. 1A–H, large arrowhead in E–H) and mature oligodendrocytes were recognized with GST-pi, which is a marker for mature oligodendrocytes (Tansey and Cammer, 1991) and Olig2, which serves as a marker of oligodendrocyte progenitor cells and mature oligodendrocytes (Zhou et al., 2000) (Fig. 1I–P, large arrowheads). Both glial cell types were lamin A-negative. Immature oligodendrocytes that were recognized with only Olig2+ staining were also negative for lamin A (Fig. 1M–P, small arrowhead). Lamin A was not observed in microglia, which were identified as being positive for ionized calcium binding adaptor molecule 1 (Iba1) (Fig. 1Q–T, large arrowheads), but observed in capillary wall cells, which were vimentin-positive (Fig. 1Q–T, small arrowheads). Iba-1 positive macrophages in the meninges were positive for lamin A (Fig. 1U–X, a small arrowhead), compared with Iba1 positive microglia in cortex (Fig. 1U–X, large arrowhead). DNA in neurons was diffusely stained by Hoechst 33258 when compared with other cells (Fig. 2I–L). Therefore, neurons that were stained with a diffuse nuclear stain were concluded to be negative for lamin A (Fig. 1A–T). We confirmed the specificity of the lamin A antibody used for multistaining by using another lamin A antibody (Millopore, MAB 3540, clone 133A2), which showed specific staining with methanol-acetone fixation (Takamori et al., 2007). Both lamin A antibodies showed identical staining patterns in the cortex (data not shown). All analysed areas showed that neural cells were negative for lamin A (Fig. 1A–D). Representative data are shown in Fig. 1 and the signal intensity of the represented images was shown in supplemental Fig. 1. There was no lamin A signal intensity in astrocytes (Fig. S1a, white arrow/bracket), mature oligodendrocytes (Fig. S1b, white allow/bracket) and microglia (Fig. S1c, a white arrow/bracket), compared with the intensity of lamin A-positive cells, such as capillary wall cells (Fig. S1a–c, yellow arrows/brackets) in the same field, which were analysed using the same microscopy imaging settings and conditions. Macrophages inside the meninges (Fig. S1d, yellow arrow/bracket) showed a lamin A-positive signal compared with microglia in the same field (Fig. S1d, white arrow/bracket). Taken together, we identified that neuron and glial cells were negative for lamin A.

### Neurons, astrocytes and mature oligodendrocytes express lamin C

**Fig. 3:**
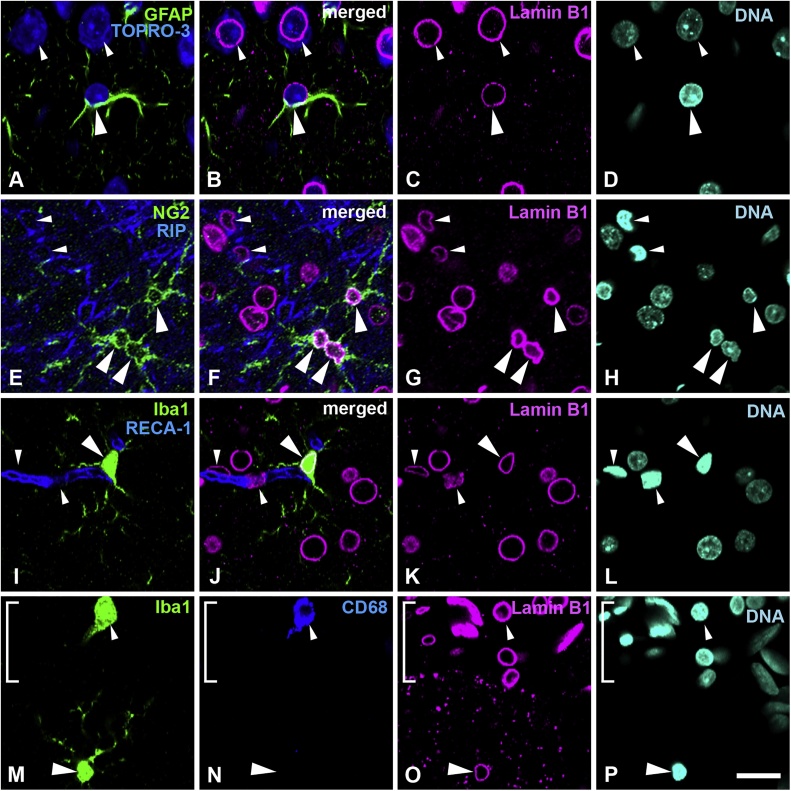
Distribution of lamin B1 in the cerebral cortex. Sections were stained with antibodies against lamin B1 (magenta) and several cell-type specific markers. A–D, Sections were fixed with 1% formaldehyde and stained with anti-GFAP antibody (green) and counterstained with TOPRO-3 for nucleic acid detection. Large arrowheads indicate astrocytes. Small arrowheads indicate neurons; also indicated by cytoplasm that is stained with TOPRO-3. E–H, Sections were stained with antibodies against NG2 (green) and RIP (blue). Large arrowheads indicate oligodendrocyte progenitor cells. Small arrowheads indicate mature oligodendrocytes. I–L, Sections were stained with antibodies against Iba-1 (green) and RECA1 (blue). Large arrowheads indicate microglia. Small arrowheads indicate capillary wall cells. M–P, Sections were stained with antibodies against Iba-1 (green) and CD68 (blue). Large arrowheads indicate microglia. Small arrowheads indicate macrophages in the meninges. The meninges were identified using brightfield imaging and shown with a white line bracket. Scale bar, 20 μm. Images of single optical sections (A-P (For interpretation of the references to colour in this figure legend, the reader is referred to the web version of this article).

**Fig. 4:**
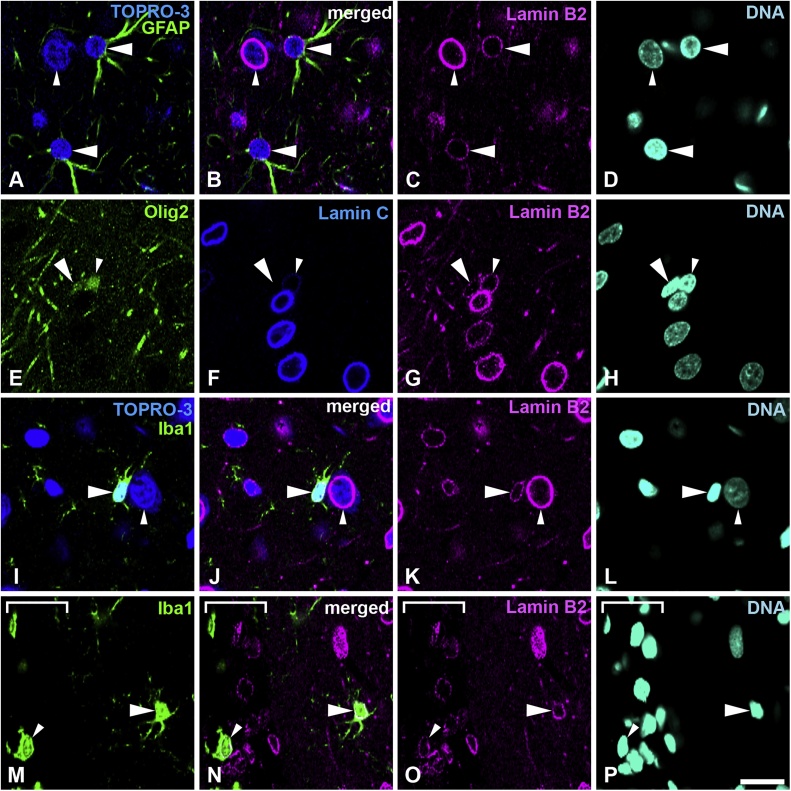
Distribution of lamin B2 in the cerebral cortex. Sections were stained with antibodies against lamin B2 (magenta) and several cell-type specific markers. A–D, Sections were stained with anti-GFAP antibody (green) and counterstained with TOPRO-3 for nucleic acid detection. Large arrowheads indicate astrocytes. Small arrowheads indicate neurons; also indicated by cytoplasm stained with TOPRO-3. E–H, Sections were stained with antibodies against Olig2 (green) and lamin C (blue). Large arrowheads indicate an oligodendrocyte progenitor cell, which is negative for lamin C. Small arrowheads indicate mature oligodendrocytes. I–L, Sections were stained with an anti-Iba-1 antibody (green) and were also counterstained with TOPRO-3 for nucleic acid detection. Large arrowheads indicate microglia. Small arrowheads indicate a neuron; these are also indicated with cytoplasm that is stained with TOPRO-3. M–P, Sections were stained with an anti-Iba-1 antibody (green). Large arrowheads indicate microglia. Small arrowheads indicate a macrophage in the meninges. The meninges were identified under bright field and shown with a white line bracket. Specimens were fixed using 4% formaldehyde combined with heat treatment. DNA was stained with Hoechst 33258 (cyan). Scale bar, 20 μm. Images of single optical sections (A–P) (For interpretation of the references to colour in this figure legend, the reader is referred to the web version of this article).

Sections were stained with rabbit anti-lamin C antibody and several cell-type specific markers to investigate the distribution of lamin C in neurons and glial cells in the cerebral cortex. Specimens were fixed using 4% formaldehyde combined with heat treatment. Lamin C was weakly positive in GFAP-positive astrocytes (Fig. 2A–D, large arrowheads) when compared with neurons (Fig. 2A–D, small arrowheads). Neurons were detected with TOPRO-3, a fluorescent dye for nucleic acid detection (Shao et al., 2011). In the cortex, TOPRO-3 stained cell nuclei and the cytoplasm of neurons (Figs. 2A–D, 3A–D, 4A–D small arrowheads) which were further recognized with a large nucleus size with diffuse irregular staining by Hoechst 33258 compared to glial cells which demonstrate small, compact and intense staining (Figs. 2A–D, 3A–D, 4A–D, large arrowheads, Matamales et al., 2009). Lamin C was observed in mature oligodendrocytes, which were intensely positive for GST-pi and positive for Olig2 (Fig. 2E–H, small arrowheads) but was not observed in immature oligodendrocyte progenitor cells, which were weakly positive for GST-pi and intensely positive for Olig2 (Fig. 2E–H, large arrowheads). In the cortex, lamin C was not observed in microglia, which were Iba-1-positive (Fig. 2I–L, large arrowheads), and differed from neurons, which were NeuN-positive (Fig. 2I–L, small arrowheads). However, lamin C was observed in macrophages, which reside in the meninges and were positive for Iba-1 (large arrowheads) (Fig. 2M–P). The staining patterns of the anti-lamin A/C antibody, which is expected to recognize both lamin A and C, purchased from Chemicon (MAB 3538, clone 131C3), and used in our previous study (Takamori et al., 2007), were like those seen using the anti-lamin C specific antibody, but not the anti-lamin A specific antibody in this study. The staining patterns shown by the anti-lamin A/C antibody in the brain might originate from lamin C (data not shown). All analysed areas showed that astrocytes, mature oligodendrocytes and neurons possessed lamin C. Representative data are shown in Fig. 2 and the signal intensity of represented images is shown in supplemental Fig. 2. Lamin C signal intensity was increased in neurons (Fig. S2a, white arrow/bracket) and astrocytes (Fig. S2a, yellow arrow/bracket) that were lamin C-positive. The intensity of lamin C in neurons was higher than that observed in astrocytes (Fig. S2a). The intensity of lamin C in mature oligodendrocytes (Fig. S2b, white arrow/bracket, Olig2+/GST-pi + high) was high, while immature oligodendrocytes (Fig. S2b, yellow arrow/bracket, Olig2+/GST-pi + significantly low) did not show any lamin C signal. Microglia did not show any lamin C signal intensity (Fig. S2c, white arrow/bracket), compared with neurons which showed high lamin C intensities (Fig. S2c, yellow arrow/bracket). Macrophages in the meninges (Fig. S2d, white arrows/brackets) showed a lamin C-positive signal.

### Lamin B1 is detected in all glial cells and neurons, especially in oligodendrocyte progenitor cells

Sections of the cortex were stained with goat anti-lamin B1 antibody and several cell-type specific markers. Specimens were fixed using 1% formaldehyde. Lamin B1 was observed in astrocytes, which were GFAP-positive (Fig. 3A–D, large arrowheads), as well as in neurons (Fig. 3A–D, small arrowheads); neuron cytoplasm and nuclei are stained with TOPRO-3. Next, we examined lamin B1 expression in the oligodendrocyte lineage (Fig. 3E–H). Lamin B1 was intensely positive in oligodendrocyte progenitor cells, which were NG2-positive and RIP-negative (Fig. 3E–H, large arrowheads), compared with mature oligodendrocytes, which were RIP-positive and NG2-negative (Fig. 3E–H, small arrowheads). Lamin B1 was observed in microglia, which were Iba-1-positive (large arrowheads) (Fig. 3I–L). Capillary wall cells, which were RECA-1-positive, were also positive for lamin B1 (Fig. 3I–L, small arrowheads). Lamin B1 was observed in meningeal macrophages, which were positive both for Iba-1 and CD68 (Fig. 3M–P, small arrowheads), and was observed in microglia, which were Iba-1-positive and CD68-negative (Fig. 3M–P, large arrowheads). Another monoclonal anti-lamin B1 antibody provided by Merck Millipore (MAB 3213, clone 119D5-F1) showed similar staining patterns in the methanol-acetone fixed brains, as lamin B1-intensely positive cells were localized to the cortex (data not shown, Takamori et al., 2007). All analysed areas showed that neural cells possessed lamin B1. Representative data are shown in Fig. 3 and the signal intensity of represented images is shown in supplemental Fig. 3. Lamin B1 signal intensities in neurons (Fig. S3a, a yellow arrow/bracket) and astrocytes (Fig. S3a, white arrow/bracket) were similar. The intensity of lamin B1 staining in immature oligodendrocytes (Fig. S3b, yellow arrows/brackets, NG2+/RIP-) was higher than that observed in mature oligodendrocytes (Fig. S3b, white arrow/bracket, NG2-/RIP+). Microglia (Fig. S3c, white allow/bracket) also possessed lamin B1 signal intensity with similar levels of staining to that observed in neurons (Fig. S3c, yellow arrow/bracket) identified with dispersed nuclear staining and largely sized nuclei (Fig. 3I–L). Although macrophages in both the meninges and microglia demonstrated lamin B1 signals, the intensity observed in macrophages, which reached the maximum with 250 intensity (Fig. S3d, white arrows/brackets), was higher than that observed in microglia, which showed intensity lower than 200 intensity (Fig. S3d, yellow arrow/bracket).

### All glial cells and neurons in the cerebral cortex express lamin B2

Sections of the cortex were stained with a rabbit anti-lamin B2 antibody and several cell-type specific markers. Specimens were fixed using 4% formaldehyde combined with heat treatment. Lamin B2 was observed in GFAP-positive astrocytes (Fig. 4A–D, large arrowheads), but the staining intensity was weak when compared to that observed in neurons; neurons were identified by cytoplasm and nuclei stained with TOPRO-3 (Fig. 4A–D, small arrowheads). Next, we examined lamin B2 in the oligodendrocyte lineage (Fig. 4E–H). Lamin B2 was observed in oligodendrocyte progenitor cells that were Olig2-positive and lamin C-negative (Fig. 4E–H, large arrowheads), as well as in mature oligodendrocytes, which were both Olig2- and lamin C-positive (Fig. 4E–H, small arrowheads), as described above. Both staining intensities were weak when compared with neurons (Fig. 4A–D and E–H). Lamin B2 was observed in Iba-1-positive microglia (Fig. 4I–L, large arrowheads), but staining intensity was weak when compared with neurons; these were recognized with their cytoplasm and nuclei stained with TOPRO-3 (Fig. 4I–L, small arrowheads). Lamin B2 was observed in Iba-1-positive meningeal macrophages (Fig. 4M–P, small arrowheads) and microglia (Fig. 4M–P, large arrowheads). All analysed areas showed that neural cells possessed lamin B2. Representative data are shown in Fig. 4 and the signal intensity of represented images is shown in supplemental Fig. 4. Lamin B2 signal intensity in neurons (Fig. S4a, yellow arrow/bracket) was higher than that observed in astrocytes (Fig. S4a, white arrow/bracket). The intensity of lamin B2 in mature (Fig. S4b, white arrow/bracket) and immature oligodendrocytes (Fig. S4b, yellow allow/bracket) were similar, whereas neurons (red arrowheads) had a higher staining intensity in same field. Microglia also possessed lamin B2 signal intensity (Fig. S4c, yellow arrow/bracket), but the signal intensity was lower than that observed in neurons (Fig. S4c, white arrow/bracket). Macrophages in the meninges and microglia in the cortex (Fig. S4d, yellow/bracket and white arrow/bracket, respectively) possessed lamin B2 signal (Fig. 4M–P), with similar staining intensities.

## Discussion

In the present study, we investigated the constitution patterns for lamin-subtypes in the neural cells of the adult rat cerebral cortex. The results of lamin-subtype patterning were shown as presented in the table (Table 3). Cortical glial cells, as well as neurons, contain lamin C as a major A-type lamin compared with lamin A. Astrocytes and oligodendrocytes, both mature glial cells, showed similar staining patterns with lamin A negative (Fig. 1A–P) and lamin C positive (Fig. 2A–H). Microglia, which are also mature glial cells, showed different patterns and lacking both lamin A (Fig. 1Q–X) and C (Fig. 2I–L). -Oligodendrocyte progenitor cells, which are in immature and proliferative states, - show no lamin C immunoreactivity (Fig. 2E–H) and -intense lamin B1 immunoreactivity, compared with mature oligodendrocytes - (Fig. 3E–H). The staining intensity of lamin B2 in all glial cells was relatively weak compared with cortical neurons (Fig. 4). These data indicate that glial cells in the adult cerebral cortex showed cell type specific lamin expression patterns.

**Table 3:** Level of lamina immunoreactivity.

	OPC	oligodendrocyte	astrocyte	neuron	microglia	macrophage
Lamin A	−	−	−	−	−	+
Lamin C	−	+	+	++	−	+
Lamin B1	++	+	+	+	+	++
Lamin B2	+	+	+	++	+	+

++, highly positive; +, positive; −, negative.

Fixation method is very important for detecting the appropriate antibody specificity. In the present study, we performed multicolor immunohistochemical analysis using antibodies against lamin subtypes and cell-type specific marker proteins, which previously have been reported to be specific for each cell type (Takamori et al., 2007). To identify mature neurons, we used anti-NeuN antibody and a fluorescent dye TOPRO-3, which stains nucleic acid dispersive. Methanol–acetone fixation has been used for suitable staining to lamin. However, this condition alters other epitopes of cell-type specific marker proteins and causes problems with the antibody specific reaction (Senda et al., 2005; Takamori et al., 2007; Wakabayashi et al., 2011). Here, we examined the suitable fixative condition for multiple immunostainings for lamins and cell-type specific marker proteins (Table 1). The staining was performed with 4% formaldehyde fixation combined with heat treatment for antibodies against lamins A, C, and B2 and with 1% formaldehyde fixation for an antibody against lamin B1.

The disease gene of adult-onset ADLD was reported to be localized to chr. 5q23-q31, including gene lamin B1 duplication (Brussino et al., 2009; Marklund et al., 2006; Meijer et al., 2008; Melberg et al., 2006). Several clinical features show autonomic dysfunction, gait disturbances, postural hypotension, urinary dysfunction and demyelination in CNS (Coffeen et al., 2000; Schwankhaus et al., 1994). CNS demyelination especially is the primary pathology of ADLD; however, cellular abnormality has not been confirmed in histopathological analysis (Coffeen et al., 2000). Meanwhile, lamin B1 overexpression in oligodendrocytes led to oligodendrocyte maturation arrest (Lin and Fu, 2009). Lamin B1 overexpression by transgenic mice with bacterial artificial chromosome clone spanning also showed cognitive abnormalities, progressive motor impairment at 12 months of age and myelination defect at 24 months of age (Heng et al., 2013). Our data indicate that the expression of lamin B1 was high in oligodendrocyte progenitor cells, however, lamin B1 was repressed during oligodendrocyte differentiation and lamin C was induced in mature oligodendrocytes in the adult brain. This finding is consistent with the previous finding that lamin B1 mRNA levels are downregulated during oligodendrocyte maturation in cultured conditions (Dugas et al., 2006). The levels of mRNA and protein of lamin B1 peaked at birth or postnatal day 1 and gradually decreased from postnatal day 1 to 10 months of age (Lin et al., 2009). Therefore, these data, including our results, strongly suggest that lamin B1 regulates OPC proliferation and maintains the immature condition (Fig. 3).

In our previous studies, in two neurogenic regions of the adult brain, neuronal progenitor cells recognized by anti-PSA-NCAM and anti-doublecortin antibodies were negative for lamin C and intensely positive for lamin B1. However, mature neurons recognized by NeuN antibody were positive for lamin C and weakly positive for lamin B1 (Takamori et al., 2007, 2014). These results indicate that the expression of lamin C is increased and lamin B1 is decreased during neuronal differentiation from progenitor cells into neurons (Table 3). The alteration patterns for lamin C and B1 during neuronal differentiation are similar to those in oligodendrocyte differentiation. Conversely, the alteration pattern for lamin B2 expression is different during the differentiation process in oligodendrocytes and neurons. The lamin B2 is constantly expressed throughout oligodendrocyte maturation; meanwhile, lamin B2 is expressed strongly in mature neurons compared with the neuronal progenitor stage (Takamori et al., 2007). Therefore, lamin B2 might have some specific function only in mature neurons. Lamin B1 and B2 knockout mice died shortly after birth and lacked the capacity for neuronal migration and survival (Coffinier et al., 2010; Kim et al., 2011). Each lamin B compensation experiment with reciprocal knock-in mice could not fully compensate a loss of each lamin B during embryo development (Lee et al., 2014), suggesting that B-type lamins have a key role for neuronal development. Recently, brain tissue in Alzheimer’s disease showed the reduction of lamin B protein level, suggesting that lamin B disruption mediates neurodegeneration in tauopathies (Frost, 2016). From these reports, B-type lamin expression in neurons is indispensable for neurogenesis and neuron survival. In the same way, lamin B2 expression in oligodendrocytes might have a role for maintaining oligodendrocyte survival through continuously expressing B-type lamins.

Microglia with small delineated processes actively screening the intra neuronal space for incoming threats were negative for lamin C and differed from astrocytes and oligodendrocytes (Fig. 2). Microglia are related closely to macrophages, which originate from hematopoietic stem cells and their origin is different from neuroectodermal-derived astrocytes and oligodendrocytes in the brain (Prinz et al., 2014). However, the meningeal macrophages, which were positive for Iba1 with round morphology and positive for lamin C, differed from microglia within the brain parenchyma (Fig. 2M–P). These data indicate that microglia and macrophages have different lamin compositions despite the related origin from hematopoietic cell lineage.

Lamin C only mice, which produce lamin C but not lamin A, display as entirely healthy. Thus, lamin A might be dispensable in mice, although the phenotype of a deficiency of both lamin A and lamin C showed slow growth, muscle weakness, and death by 6 weeks of age. (Fong et al., 2006). Meanwhile, mutation of LMNA causes multiple human diseases including muscular dystrophy, cardiomyopathy, and lipodystrophy (Worman et al., 2009). A few mutations of prelamin A, which alter the structure of prelamin A, induces Hutchinson-Gilford progeria syndrome (Eriksson et al., 2003; De Sandre-Giovannoli et al., 2003). Two knock-in lines contained a 5-nucleotide mutation in the miR-9 binding site in prelamin A 3’UTR, and replaced prelamin A 3’UTR with lamin C 3’UTR, induced large amounts of lamin A in the brain, and pathological defects were detected mainly in capillary endothelial cells. However, there was no obvious neuropathology or behavior disorder, which could explain the absence of abnormal pathology in CNS (Jung et al., 2014). Our data, in which lamin A was not observed in the neural cells in the cerebral cortex but was observed in capillary wall cells, confirmed that lamin A might not be relate directly to CNS, but there might be indirect relations via endothelial cells in the brain parenchyma and meninges.

In summary, our study showed the composition of each lamin subtype in the glial cells of adult brain using an immunohistochemical procedure. Current information might be useful to understand the onset mechanism of human neurodegenerative diseases associated with nuclear lamina proteins as well as functional differences of each lamin subtypes.

## Author contributions

Y.T. performed tissue preparation, immunostaining and the data analysis and wrote the manuscript. T.W. and T.M. T.K. contributed to data collection and analysis. Y.K., Y.T., S.K., K. K., took part in the data analysis and gave suggestions. H.Y. supervised the study. Y.H. supervised the study and wrote the manuscript. All authors read and approved the final manuscript.

## Conflicts of interest

The authors declare no competing financial interests.

## Figures and Tables

**Figure:**
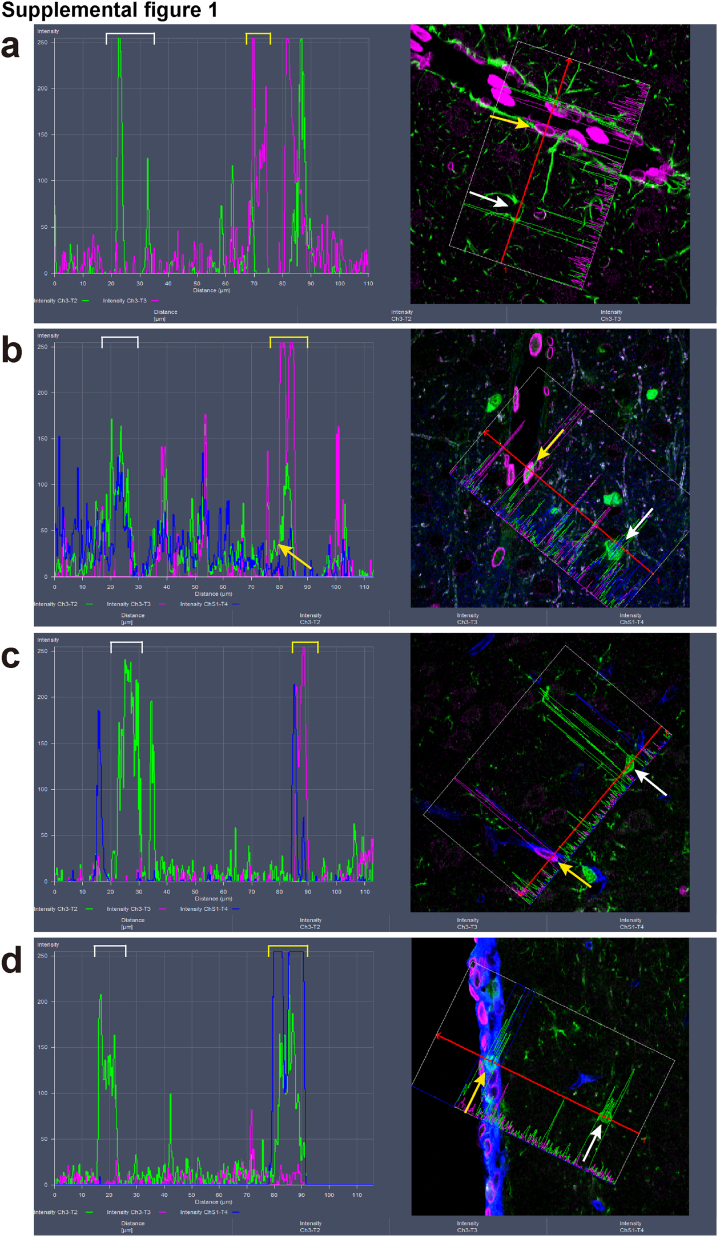


**Figure:**
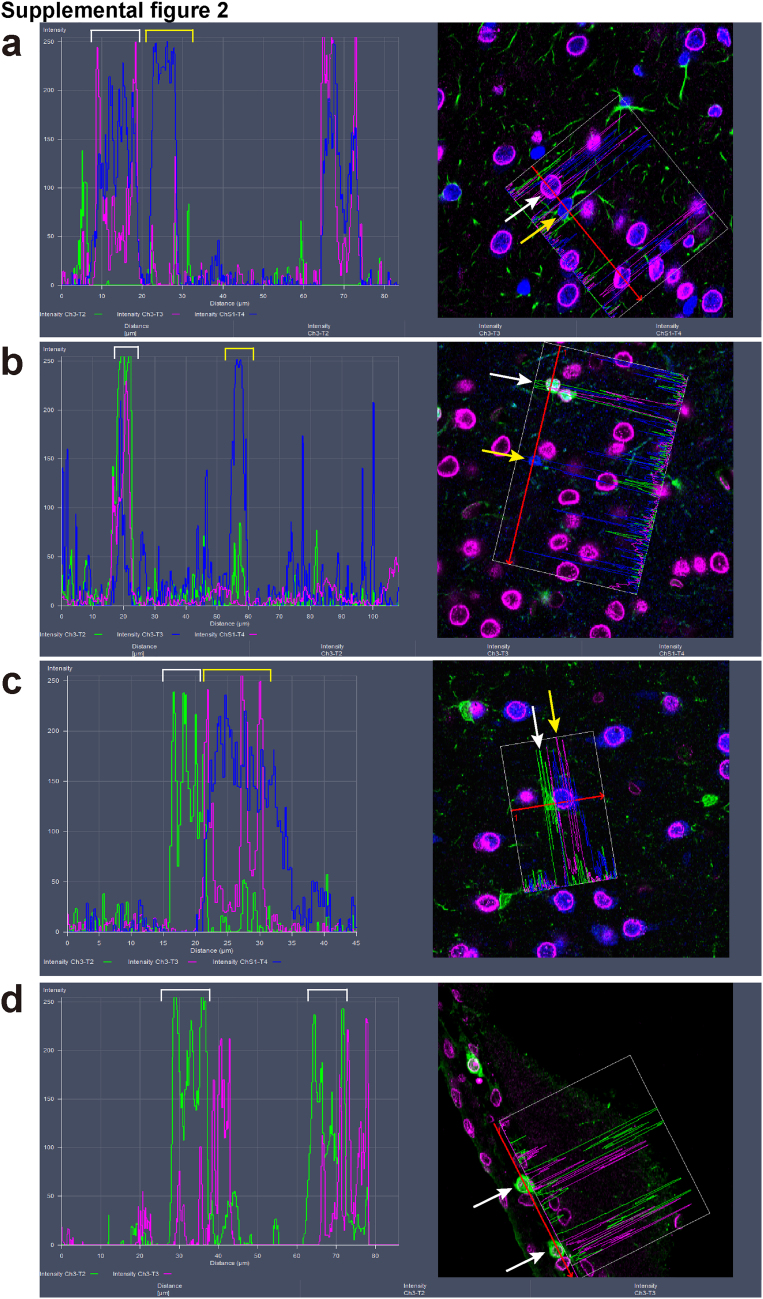


**Figure:**
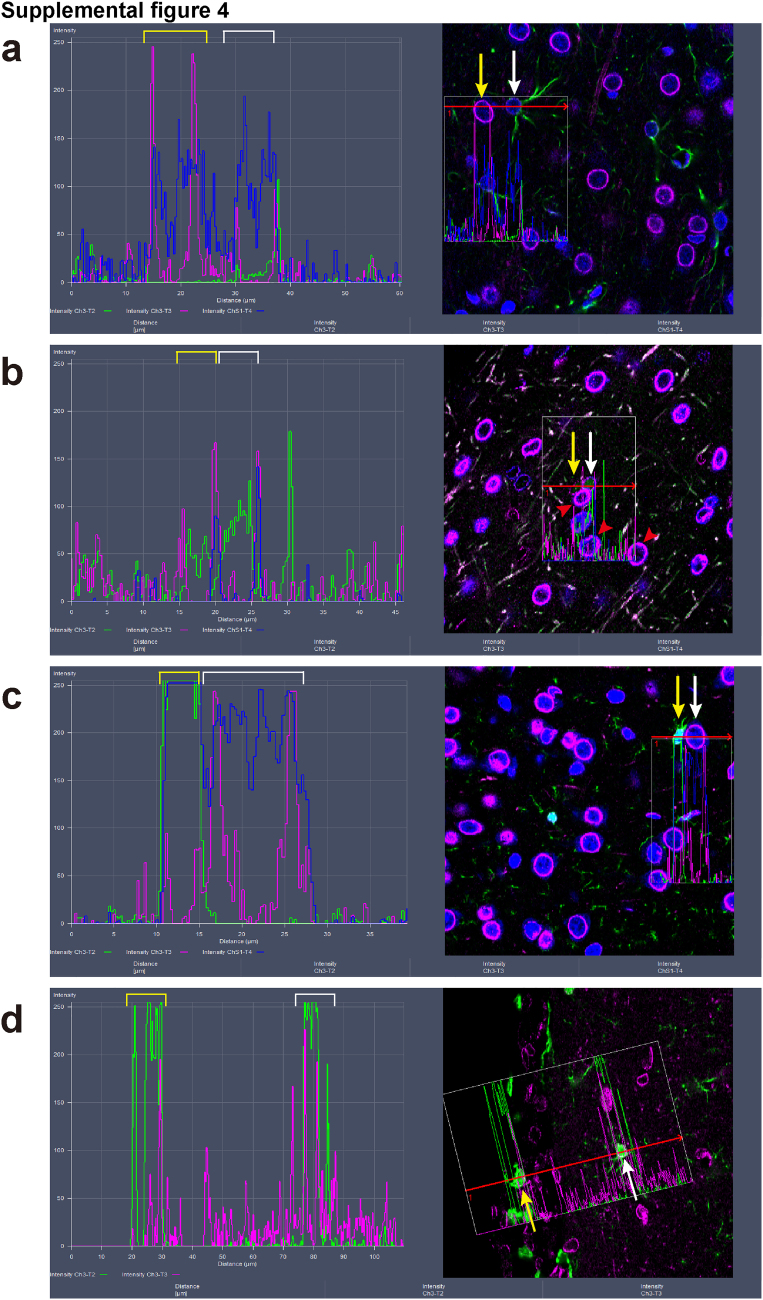


**Figure:**